# Mutations in Animal SARS-CoV-2 Induce Mismatches with the Diagnostic PCR Assays

**DOI:** 10.3390/pathogens10030371

**Published:** 2021-03-19

**Authors:** Ahmed Elaswad, Mohamed Fawzy

**Affiliations:** 1 Department of Animal Wealth Development, Faculty of Veterinary Medicine, Suez Canal University, Ismailia 41522, Egypt; 2 Department of Virology, Faculty of Veterinary Medicine, Suez Canal University, Ismailia 41522, Egypt; 3 Middle East for Vaccines (ME VAC®), Sharquia 44813, Egypt

**Keywords:** coronavirus, COVID-19, diagnostic assay, mutations, mismatches, PCR

## Abstract

Recently, the severe acute respiratory syndrome coronavirus-2 (SARS-CoV-2) was detected in several animal species. After transmission to animals, the virus accumulates mutations in its genome as adaptation to the new animal host progresses. Therefore, we investigated whether these mutations result in mismatches with the diagnostic PCR assays and suggested proper modifications to the oligo sequences accordingly. A comprehensive bioinformatic analysis was conducted using 28 diagnostic PCR assays and 793 publicly available SARS-CoV-2 genomes isolated from animals. Sixteen out of the investigated 28 PCR assays displayed at least one mismatch with their targets at the 0.5% threshold. Mismatches were detected in seven, two, two, and six assays targeting the ORF1ab, spike, envelope, and nucleocapsid genes, respectively. Several of these mismatches, such as the deletions and mismatches at the 3’ end of the primer or probe, are expected to negatively affect the diagnostic PCR assays resulting in false-negative results. The modifications to the oligo sequences should result in stronger template binding by the oligos, better sensitivity of the assays, and higher confidence in the result. It is necessary to monitor the targets of diagnostic PCR assays for any future mutations that may occur as the virus continues to evolve in animals.

## 1. Introduction

The global outbreak of coronavirus disease-2019 (COVID-19) caused by the severe acute respiratory syndrome coronavirus-2 (SARS-CoV-2) was first reported in Wuhan city, Hubei province, China in December 2019 [1,2]. It was announced by the World Health Organization (WHO) as a public health emergency of international concern then identified as a pandemic disease on 11 March 2020. The number of confirmed cases has been rising dramatically; as of 16 March 2021, the virus had spread to 219 countries and territories with around 120 million confirmed cases and 2.6 million deaths. The pandemic spread of the virus is related to its transmission by the symptomatic and asymptomatic carriers with the presence of animal reservoirs [3,4].

SARS-CoV-2 is an enveloped, single-stranded, positive-sense RNA virus that belongs to the family *Coronaviridae*, subfamily *Orthocoronavirinae*, and genus *Betacoronavirus*. The genome is 29,903 nucleotides in size that encodes 16 non-structural proteins (nsp1-nsp16), 6 accessory proteins (3a, 6, 7a, 7b, 8, and 10), and four structural proteins (S, spike; E, envelope; M, matrix; and N, nucleocapsid) [5].

The rapid diagnosis of SARS-CoV-2 infection is the cornerstone for policymakers to control the outbreak. The scheme of COVID-19 diagnosis depends on epidemiological history, laboratory diagnosis, virus isolation, serological identification, molecular confirmation, and radiological diagnosis [6]. SARS-CoV-2 nucleic acid detection is the main, most specific, sensitive, and rapid tool for diagnosis of the infection [7]. Therefore, the WHO recommended the reverse transcription–quantitative polymerase chain reaction (RT–qPCR) as a gold standard method for SARS-CoV-2 identification [8]. Consequently, several PCR detection assays have been developed for this purpose [1,9,10,11,12,13,14,15,16,17,18]. The accuracy of PCR detection can be influenced by several factors including primer/probe design [19], sample impurities [20], non-specific annealing [21], cross-reactivity with other viruses [13], reagent contamination [22], poor amplification efficiency [23], and hybridization melting temperature [24].

Despite the presence of an RNA proofreading exoribonuclease (nsp14-ExoN) [25], the circulating SARS-CoV-2 genome exhibited several mutations either in humans or animals [26,27]. These mutations may lead to mismatches if occurred at primer or probe binding regions, resulting in false-negative results [19]. Moreover, single-nucleotide mismatches may affect only the first few cycles of PCR, but with an appropriate design, the detection of the target may not be affected [28]. Improper diagnosis due to primer/probe mismatches has been reported for several viruses including SARS-CoV-2 [29], dengue virus [30], hepatitis B virus [31,32], human immunodeficiency virus [33], influenza virus [34], rabies virus [35], and respiratory syncytial virus [36].

The first recorded cases of COVID-19 were associated with the Huanan Seafood Wholesale Market in the Wuhan province of China, suggesting transmission of the disease from animals [1]. Bats may be considered a potential reservoir host to SARS-CoV-2 due to the high identity (96.3%) with bat coronavirus RaTG13 [2]. SARS-CoV-2 has been isolated from several animal hosts in many countries [4,37]. SARS-CoV-2 has been identified in dogs in Hong Kong and the United States, where viral sequences from dogs in Hong Kong were identical to those isolated from the respective human cases, suggesting human-to-animal transmission [38]. SARS-CoV-2 was also detected in cats from several countries including Belgium, Chile, Denmark, England, France, Greece, Hong Kong, Spain, and the United States [39,40,41,42,43]. In addition, SARS-CoV-2 was identified in lions and tigers in a zoo in New York, United States [44]. Experimental infection was achieved in golden Syrian hamsters (*Mesocricetus auratus*) via oral and intranasal routes [45]. Beginning April and May 2020, outbreaks of SARS-CoV-2 infection were reported in American and European mink (*Neovison vison* and *Mustela lutreola*, respectively) farms in the Netherlands and Denmark [46,47,48]. In these outbreaks, the genomic signature of SARS-CoV-2 isolated from workers in mink farms was identical to that of animal sequences, supporting the evidence of animal-to-human transmission of SARS-CoV-2 in mink farms [47]. Therefore, control of SARS-CoV-2 in animals is crucial to control the disease in humans.

After cross-species transmission, the virus begins to acquire mutations to adapt to the new hosts, which may result in new viral strains [49]. We previously reported several unique mutations in SARS-CoV-2 isolated from cats, dogs, minks, and mice when compared with SARS-CoV-2 isolates from humans at the same time and geographic region [26]. These acquired nucleotide variations may occur all over the genome including the targets of diagnostic PCR assays. Depending on the nature of on-target mutations, the sensitivity of diagnostic PCR assays may be affected. The currently available diagnostic PCR assays were initially developed for detecting SARS-CoV-2 in humans. Perfect matches between the primer/probe binding regions would increase the sensitivity of the diagnostic tests and reduce the occurrence of false-negative results. Therefore, the objectives of the current study were (1) the in-silico reassessment of currently available diagnostic PCR primers and probes for detecting SARS-CoV-2 in animal hosts and (2) suggesting modifications to the primers or probe sequences depending on the mutations identified in animal isolates. 

## 2. Materials and Methods

### 2.1. Selection of SARS-CoV-2 Genomes

A total of 793 SARS-CoV-2 genomes isolated from animals were used in the current study. These were all the available animal SARS-CoV-2 genomes from the Global Initiative on Sharing All Influenza Data (GISAID) and the National Center for Biotechnology Information (NCBI) databases as of 10 January 2021. SARS-CoV-2 reference genome (NC_045512.2, Wuhan-Hu-1 isolate) was also downloaded and included in the analysis. The 793 SARS-CoV-2 animal genomes were all complete (>29,000 nucleotides) except one genome from the dog (EPI_ISL_414518, 27,871 nucleotides). The genomes originated from seven different animal species and 13 geographic regions (Table 1). They included 19 from the cat, five from the dog, five from the golden hamster (*Mesocricetus auratus*), four from the lion, 753 from the mink, six from the tiger, and one from the mouse. Animal SARS-CoV-2 genomes were submitted from Asia (9 genomes), Europe (762), North America (18), and South America (4). Information on SARS-CoV-2 genomes used in the current study can be found in Table S1. This information includes the virus isolate, accession number, host, geographic region or country, genome length, collection date, database from which they were downloaded, and the percentage of ambiguous bases (%N).

### 2.2. Selection of Diagnostic PCR Assays

A total of 28 primer-probe set binding sites were investigated in the current study (Table 2). They included primer-probe sets from assays listed on the World Health Organization (WHO) website [15] and developed by the Chinese Center for Disease Control and Prevention (China CDC), China; the Centers for Disease Control and Prevention, Atlanta, GA, United States (US CDC); the Institute of Virology—Charité—Universitätsmedizin Berlin, Germany; the National Institute of Infectious Diseases (NIID), Japan; Institute Pasteur, Paris, France; The University of Hong Kong (HKU), Hong Kong; and the National Institute of Health of Thailand (THAI NIH), Thailand; in addition to several other assays developed by researchers [1,9,10,11,12,13,14,16,17,18]. 

**Figure 1:**
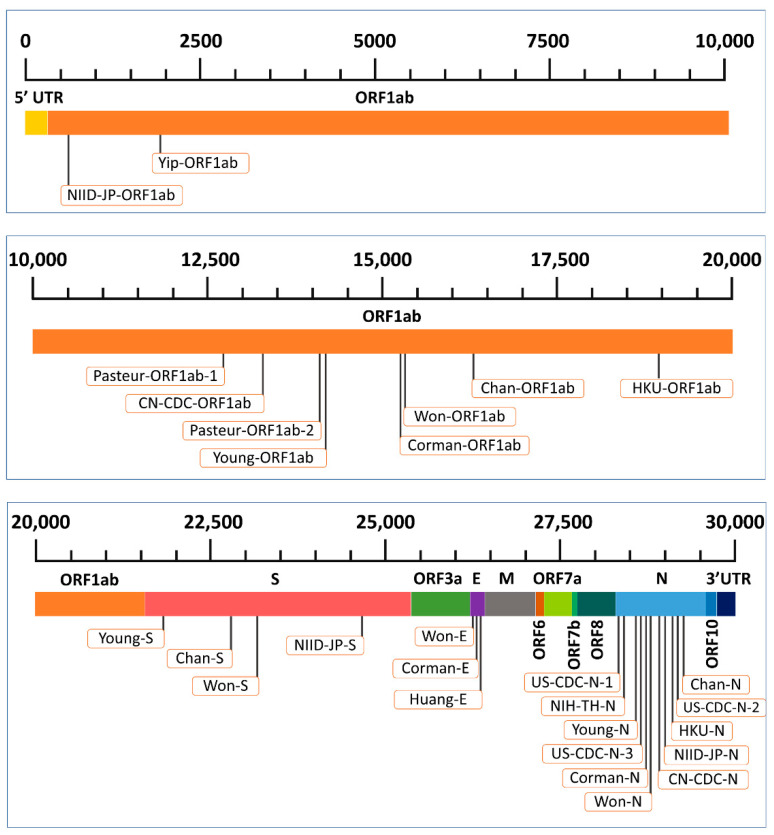
Representation of the genomic targets of the current diagnostic PCR assays in animal SARS-CoV-2 genome.

The distribution of the 28 PCR assays along the SARS-CoV-2 genome was as follows: 10 in the ORF1ab gene, four in the S gene, three in the E gene, and 11 in the N gene (Figure 1). The assays were named in the current study depending on the developing organization or researcher and following [19]. For example, CN-CDC-ORF1ab was developed by the Chinese Center for Disease Control and Prevention for the ORF1ab gene. Similarly, the Young-S assay was developed by Young et al. [18] for the S gene.

### 2.3. Multiple-Sequence Alignment

All animal sequences and the reference sequence were aligned using Multiple Sequence Comparison by Log-Expectation (MUSCLE) v3.8.31 [50]. The quality of the multiple sequence alignment (MSA) results was checked in AliView [51]. Edits to the alignment were manually introduced when necessary to obtain the best alignment. The MSA length was 29,903 (the same length as the reference genome), and the nucleotide positions in all genomes were called based on the positions in the reference genome. The MSA was exported in FASTA format. 

### 2.4. Identification of Nucleotide Changes at the Primer-Probe Binding Sites

**Figure 2:**
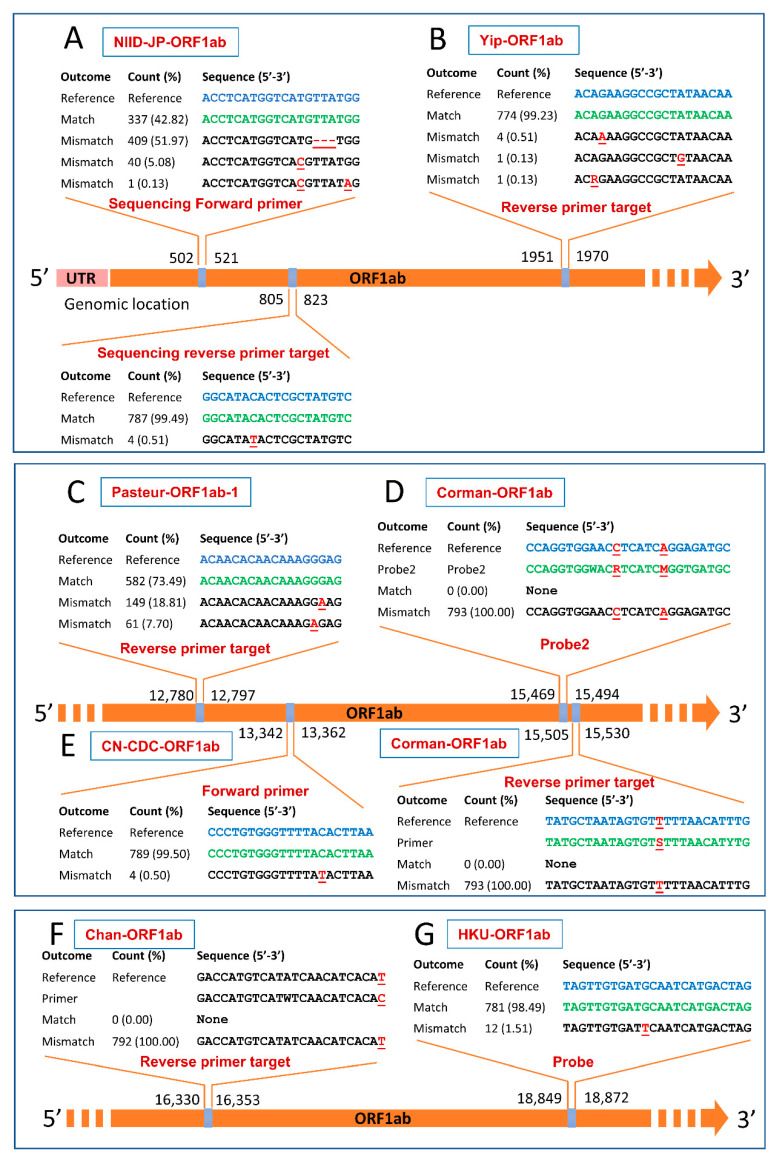
Mismatches in the primer/probe targets of diagnostic PCR assays targeting open reading frame 1ab (ORF1ab) gene of animal SARS-CoV-2. Perfect matches, mismatches, and nucleotide deletions are represented by green letters, red (underlined) letters, and red (underlined) dashes, respectively. Reverse primers are reverse complemented. Numbers and percentages here are calculated based on the informative sequences only, and non-informative (ambiguous) sequences were excluded. Refer to the Materials and Methods for information on the nomenclature of the assays illustrated in this figure.

**Figure 3:**
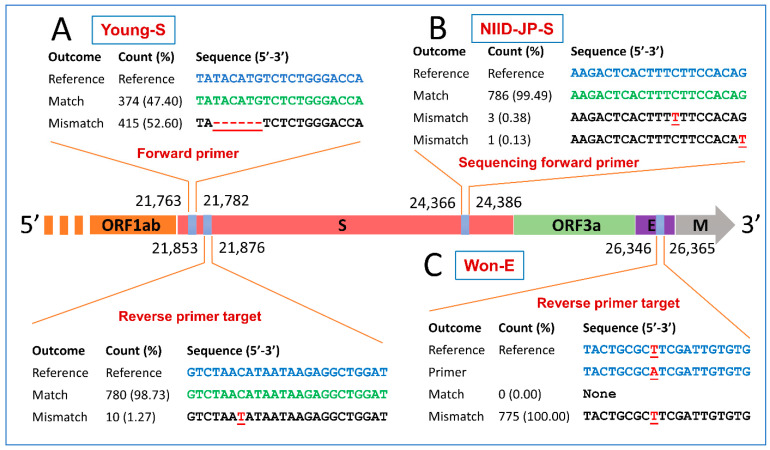
Mismatches in the primer/probe targets of diagnostic PCR assays targeting the spike (S) and envelope (E) genes of animal SARS-CoV-2. Perfect matches, mismatches, and nucleotide deletions are represented by green letters, red (underlined) letters, and red (underlined) dashes, respectively. Reverse primers are reverse complemented. Numbers and percentages here are calculated based on the informative sequences only, and non-informative (ambiguous) sequences were excluded. Refer to the Materials and Methods for information on the nomenclature of the assays illustrated in this figure.

**Figure 4:**
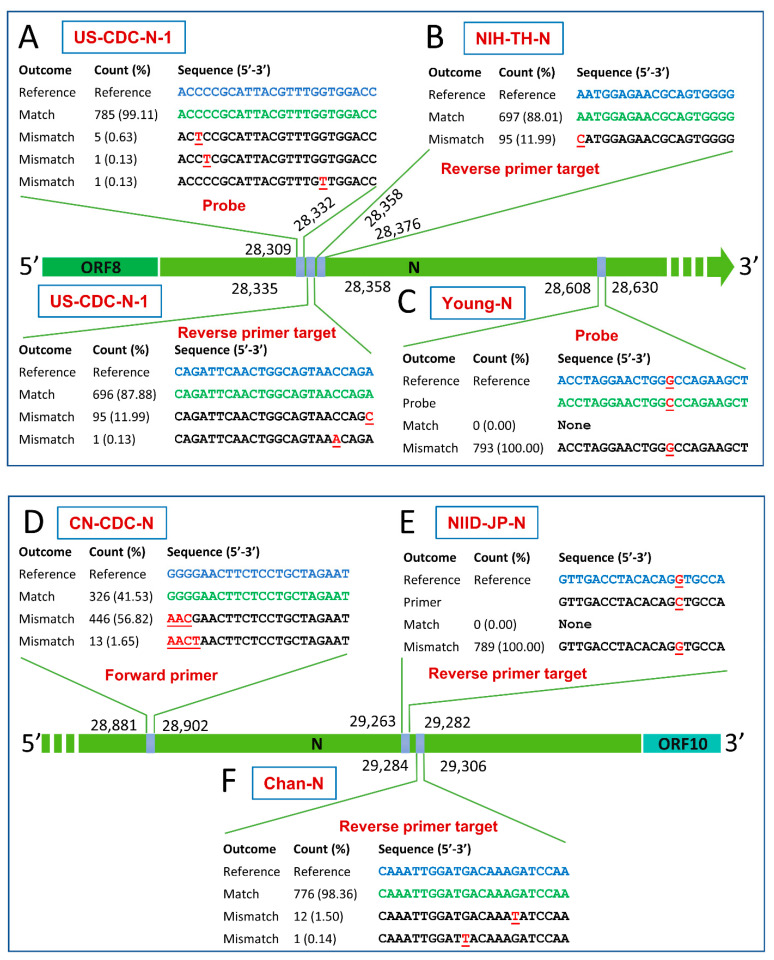
Mismatches in the primer/probe targets of diagnostic PCR assays targeting the nucleocapsid (N) gene of animal SARS-CoV-2. Perfect matches and mismatches are represented by green and red (underlined) letters, respectively. Reverse primers are reverse complemented. Numbers and percentages here are calculated based on the informative sequences only and non-informative (ambiguous) sequences were excluded. Refer to the Materials and Methods for information on the nomenclature of the assays illustrated in this figure.

In all the analyses, reverse primers were reverse complemented, and the mutations were investigated at the binding sites in the MSA. The same was performed for the probe designed by HKU for the N gene (HKU-N) as it was an antisense probe. Nucleotide variations at the primer/probe sequences or binding sites were investigated in AliView. Sequences with at least one ambiguous nucleotide (N) at any binding site were excluded for that binding site. The analysis results are reported in Table S2. To exclude the sequencing errors and infrequent mutations, a threshold of 0.5% [19] was applied in reporting the nucleotide variation. In this case, variations that existed in less than four genomes were considered below the 0.5% threshold and therefore not reported here in the main Tables or Figures (reported only in Table S2). When the variations were above the threshold, the sequences at the binding site of a primer/probe were exported in FASTA format and stratified using the Sequence Tracer module of the Alignment Explorer (Available online at http://entropy.szu.cz:8080/EntropyCalcWeb/sequences (accessed on 15 January 2021)). This module sorts the identical sequence variants into discrete groups and calculates their frequencies. The results of the Sequence Tracer module are presented in Figure 2, Figure 3 and Figure 4 for the ORF1ab, S and E, and N genes, respectively. 

## 3. Results

A total of 793 SARS-CoV-2 animals’ genomes isolated from cats, dogs, golden hamsters, lions, minks, tigers, and mouse were used in this study. Twelve out of the investigated 28 PCR assays displayed a perfect match with their targets at the determined threshold. The detailed information on assay names, countries, animal species, primer/probe sequences, positions, number of match and mismatch nucleotides are available in Table S2.

### 3.1. Mismatches in Diagnostic PCR Assays Targeting the ORF1ab Gene

It was observed that out of the 10 assays targeting the ORF1ab gene, three showed a perfect match with animal isolates at the defined threshold. These three assays were the Pasteur-ORF1ab-2, Young-ORF1ab, and Won-ORF1ab. The NIID-JP-ORF1ab had mismatches for the two sequencing primers (forward and reverse). Mismatches for the forward sequencing primer occurred at a total frequency of 57.2% including three nucleotide deletions (51.97%), one nucleotide mismatch (5.08%), and two nucleotide mismatches (0.13%). The reverse sequencing primer displayed a single mismatch with 0.51% of animal sequences as shown in Figure 2A. The reverse primer of Yip-ORF1ab displayed a single-nucleotide substitution with 0.77% of analyzed sequences (Figure 2B). In Pasteur-ORF1ab-1, the forward primer and the probe displayed a perfect match with all the studied genomes (100%), while only 582 of 792 informative sequences (73.48%) had a perfect match with the reverse primer. The remaining sequences (210) exhibited two types of single mismatches (Figure 2C). In Corman-ORF1ab, probe2 displayed two nucleotide substitutions (C-R and A-M) with all sequences, while the reverse primer showed one mismatch (T-S) with all tested animal sequences (Figure 2D). The forward primer and probe1 of Corman-ORF1ab perfectly matched all the studied informative sequences. The CN-CDC-ORF1ab forward primer displayed a single mismatch with 0.5% of the sequences, as illustrated in Figure 2E. One mismatch (T-C) was also observed with all tested animals’ sequences for the reverse primer of the Chan-ORF1ab assay (Figure 2F). The HKU-ORF1ab probe showed a single mismatch with 1.51% of sequences (Figure 2G).

### 3.2. Mismatches in Diagnostic PCR Assays Targeting the S Gene

Out of the four investigated PCR assays for the S gene, Chan-S and Won-S perfectly matched the studied genomes at the 0.5% threshold. Mismatches were observed for the forward and reverse primers of the Young-S assay and the sequencing forward primer of the NIID-JP-S assay. The forward primer of the Young-S assay perfectly matched with 374 sequences (47.4%), while mismatches occurred in 415 sequences (52.60%) due to a deletion of six nucleotides (TACATG). The reverse primer of Young-S showed one nucleotide mismatch with 1.27% of sequences, as shown in Figure 3A. The sequencing forward primer of the NIID-JP-S assay showed a perfect match with 99.49% of sequences and two types of single-nucleotide mismatches with 0.51% of animal sequences (Figure 3B).

### 3.3. Mismatches in Diagnostic PCR Assays Targeting the E Gene

Two out of the three tested PCR assays targeting the E gene perfectly matched the studied genomes at the defined threshold. The reverse primer of the Won-E assay exhibited a single-nucleotide substitution (A-T) with all tested viral sequences as shown in Figure 3C. 

### 3.4. Mismatches in Diagnostic PCR Assays Targeting the N Gene

It was observed that, out of the investigated eleven assays targeting the N gene, five assays (US-CDC-N-2, US-CDC-N-3, Corman-N, Won-N, and HKU-N) displayed a perfect match with the studied genomes at the determined threshold. The US-CDC-N-1 probe and reverse primer showed single-nucleotide mismatches with 0.89% and 12.12% of animals’ sequences, respectively, as demonstrated in Figure 4A. The reverse primer of NIH-TH-N assay matched 697 sequences and mismatched 95 tested sequences with a percentage of 88.01% and 11.99%, respectively (Figure 4B). One mismatch (C-G) was observed with all animal sequences for the Young-N probe (Figure 4C). The forward primer of the CN-CDC-N assay displayed three and four nucleotide mismatches with 56.82% and 1.65% of sequences, respectively (Figure 4D). In addition, the NIID-JP-N reverse primer showed a single-nucleotide mismatch (G-C) with all tested sequences (Figure 4E). The reverse primer of Chan-N showed two single mismatches with 1.64% of sequences as observed in Figure 4F.

### 3.5. Suggested Modifications of Primer-Probe Sets

Based on the reported variations at the primer-probe binding sites, we suggested some adjustments to the primer-probe sequences using the International Union of Pure and Applied Chemistry (IUPAC) nucleotide codes (Table 3). These adjustments were performed for the mismatches above the threshold. 

## 4. Discussion

Our study aimed to evaluate the currently available diagnostic PCR primers and probes, either recommended by WHO or published in the latest literature, for the detection of SARS-CoV-2 in animal hosts. We identified potential mutations at the primer/probe binding sites in SARS-CoV-2 isolated from animals and suggested several modifications to the primers and probe sequences to perfectly match their targets. Perfect match between PCR oligos and their targets will increase the confidence in the results and help veterinarians, technicians, laboratory professionals, clinicians, and policymakers control the disease in animals and humans. To this extent, 28 diagnostic PCR assays were in silico evaluated using 793 SARS-CoV-2 genomes isolated from cats, dogs, golden hamsters, lions, minks, tigers, and mouse. To prevent any bias in methodology, several points were considered. (1) All animal SARS-CoV-2 genomes available from the GISAID and NCBI databases from various geographical regions (Asia, Europe, North America, and South America) were selected for reassessment of the assays. (2) The MSA length was 29,903, which is the same length as the reference genome, and the short sequences were not included in our analysis. (3) Sequences with at least one ambiguous nucleotide (N) at any binding site were omitted. (4) In the reporting of nucleotide variation, a threshold of 0.5% was applied to remove sequencing errors and infrequent mutations. (5) Using the Sequence Tracer module allows incomplete or short sequences to be filtered out, identical sequence variants to be sorted into different classes, and their frequencies to be determined.

In this study, sixteen out of the investigated 28 PCR assays displayed at least one mismatch with their templates. This number is higher than that obtained by [19] who reported mismatches in seven out of 27 assays. This result may be due to the ongoing adaptation of SARS-CoV-2 in animal hosts resulting in higher variations in animal isolates compared with human isolates [26]. These variations highlighted the need for frequent evaluation of currently available diagnostic PCR assays to successfully control the SARS-CoV-2 pandemic. On the other hand, twelve out of the 28 PCR assays showed a perfect match with their targets at the determined threshold. These findings may be supported by the lower mutation rates in coronaviruses compared with other RNA viruses due to the RNA proofreading activity of nsp14-exoribonuclease [25,52]. In case of SARS-CoV-2, the virus acquires two mutations in its genome per month with an estimated evolutionary rate of 1.15 × 10^−3^ substitutions/site/year [53,54].

Several mismatches with the investigated PCR assays were reported. These mismatches were not necessary to produce false-negative results as the effect of the mismatch varied according to the number, positions, and target (probe, forward, or reverse primer). The negative effect of a single-nucleotide mismatch on target annealing is lower than deletions or multiple-nucleotide mismatches. Mismatches near the 3′ end can affect the target’s amplification and detection while a single mismatch located near the 5′ end or more than five bases from the 3′ end can affect only the first few PCR cycles with no noticeable impact on the amplification process [55,56,57]. Single mismatches in the reverse or forward primers may not have a significant impact on target detection. However, a single mismatch in the probe may result in a false-negative, as it prevents the probe binding and fluorescence emission [32,36,58,59].

In our study, (1) Single-nucleotide mismatches were reported near the 3′ end in NIID-JP-ORF1ab sequencing forward primer, Pasteur-ORF1ab-1 reverse primer, Chan-ORF1ab reverse primer, NIID-JP-S sequencing forward primer, and US-CDC-N-1 reverse primer, (2) Fatal deletions were detected in two assays: NIID-JP-ORF1ab sequencing forward primer and Young-S forward primer, (3) Multiple-nucleotide mismatches were observed in NIID-JP-ORF1ab sequencing forward primer, Corman-ORF1ab probe2, and CN-CDC-N forward primer, (4) Mismatches in probes that may result in false-negative were detected in four assays: Corman-ORF1ab, HKU-ORF1ab, US-CDC-N-1, and Young-N, and (5) A single mismatch with all animal sequences was observed in Corman-ORF1ab probe2, Corman-ORF1ab reverse primer, Chan-ORF1ab reverse primer, Won-E reverse primer, Young-N probe, and NIID-JP-N reverse primer. Shirato and his colleagues then updated the NIID-JP-N reverse primer to correct such mismatch in another report [14]. Mismatches in the Corman-ORF1ab probe2 were introduced by the authors so that the probe2 detects SARS-CoV-2, SARS-CoV, and bat-SARS-related CoVs [12]. The amplification method might not be influenced by a single mismatch near the 5’ end; however, correction of such mismatches would ensure stronger template binding, better sensitivity, and higher confidence in the results. Therefore, we suggested several modifications to the oligos that did not perfectly match SARS-CoV-2 genomes from animals (Table 3). However, the proposed modifications may require experimental testing using COVID-19 confirmed clinical samples considering the low sensitivity of certain diagnostic PCR assays in some cases [60,61].

It was observed that three (Pasteur-ORF1ab-2, Young-ORF1ab, and Won-ORF1ab) of the 10 assays targeting the ORF1ab gene showed a perfect match with animal isolates at the specified threshold. These findings are in agreement with Khan and Cheung [19], who used 17,175 human SARS-CoV-2 sequences to test the three assays. Seven out of 9 assays targeting the ORF1ab gene showed a perfect match with human SARS-CoV-2 isolates in the study conducted by Khan and Cheung [19], and only two (Chan-ORF1ab probe and Charite-ORF1b reverse primer) showed a mismatch at the same threshold. Compared to the previous study [19], the higher number of mismatches in our study (seven) may be attributable to the mutations investigated in ORF1ab of SARS-CoV-2 animal genomes [26]. Positive selection has also been demonstrated for specific residues of the non-structural proteins of ORF1ab and the accessory proteins ORF3a and ORF8. These sites of the SARS-CoV-2 genome may be significant in generating variants adapted to humans or animals. Such findings can affect the production of diagnostic tests, therapeutics and preventive instruments, such as vaccines and antivirals [54].

In our study, we reported mismatches in one (Won-E) of the current three assays targeting the E gene compared to none reported by Khan and Cheung [19]. For the assays targeting the N gene, we revealed mismatches in six (US-CDC-N-1, NIIH-TH-N, Young-N, CN-CDC-N, NIID-JP-N, and Chan-N) out of eleven assays compared to five (CN-CDC-N, US-CDC-N-1, US-CDC-N-3, Young-N, and NIID-JP-N) out of eleven observed by Khan and Cheung [19]. The N and E genes encode essential coronavirus capsid structural proteins, while other proteins regulate a range of molecular processes during viral replication [62]. The E gene is highly conserved with no mutations [26]. The single-nucleotide mismatch observed here in Won-E reverse primer is likely due to the primer design, not the evolution of animal SARS-CoV-2 at this site, because this mismatch is present in all the studied genomes including the reference sequence (Wuhan-Hu-1). The N gene may be under positive selective pressure where it is accumulating a significant number of mutations in human and animal isolates [26,63].

At the 0.5% threshold, two of the four investigated PCR assays targeting the S gene (Young-S forward and reverse primers, and NIID-JP-S sequencing forward primer) displayed mismatches with the studied genomes. On the contrary, Khan and Cheung [19] did not find any nucleotide mismatches in the assays targeting the S gene. The SARS-CoV-2 spike protein plays a major role in host cell receptor attachment, neutralizing antibody production, and host tropism allocation [2]. The SARS-CoV-2, like SARS-CoV and HCoV-NL63, uses the angiotensin-converting enzyme 2 (ACE2) receptor for host cell entry [2,64,65]. As the virus infects the animals and evolves during the outbreak, nucleotide substitutions may emerge in the primer/probe binding regions including the S gene [54,66,67]. The current SARS-CoV-2 genomes were isolated from animals where there are considerable differences in the ACE2 receptors compared with humans. Therefore, adaptation of the virus to animals will likely be different from humans, resulting in the accumulation of different mutations in the S gene due to the differences in the ACE2 [26,68,69]. The S gene was reported to be under persistent positive selection [66], which may result in additional mutations accumulating in the S gene in the future.

## 5. Conclusions

We evaluated 28 diagnostic PCR assays that were initially developed to detect SARS-CoV-2 in humans, for the detection of SARS-CoV-2 in animals. Sixteen out of the investigated 28 PCR assays displayed at least one mismatch with their targets at the 0.5% threshold. These mismatches were attributed to the continuous evolution occurring in SARS-CoV-2 in animals. Several of these mismatches are expected to negatively affect the diagnostic PCR assays. Therefore, we suggested some modifications to the oligo sequences accordingly. These suggestions should result in stronger template binding by the oligos, better sensitivity of the assays, and higher confidence in the results. As the virus continues to evolve in animals and accumulates mutations in its genome, it is crucial to frequently monitor the effects of these mutations on the diagnostic PCR assays and modify them accordingly. This should reduce the probability of false-negative results and help control the COVID-19 pandemic in animals and humans.

## Figures and Tables

**Table 1:** Numbers of animal SARS-CoV-2 genomes used in the current study.

Continent	Country	Host Species								
		American Mink	Cat	Dog	European Mink	Golden Hamster	Lion	Mouse	Tiger	Total
Asia	China	-	-	-	-	-	-	1	-	1
	Hong Kong	-	1	2	-	5	-	-	-	8
Europe	Belgium	-	1	-	-	-	-	-	-	1
	Denmark	454	3	-	-	-	-	-	-	457
	England	-	1	-	-	-	-	-	-	1
	France	-	3	-	-	-	-	-	-	3
	Greece	-	1	-	-	-	-	-	-	1
	Italy	-	-	1	-	-	-	-	-	1
	Netherlands	270	1	1	13	-	-	-	-	285
	Poland	12	-	-	-	-	-	-	-	12
	Spain	-	1	-	-	-	-	-	-	1
North America	Canada	4	-	-	-	-	-	-	-	4
	USA	-	3	1	-	-	4	-	6	14
South America	Chile	-	4	-	-	-	-	-	-	4
Total	Total	740	19	5	13	5	4	1	6	793

**Table 2:** Information on the 28 SARS-CoV-2 diagnostic PCR assays investigated in the current study.

Assay	Country	Oligo	Sequence (5’-3’)	Genome Position	Reference
**ORF1ab**					
NIID-JP-ORF1ab	Japan	F1	TTCGGATGCTCGAACTGCACC	484–504	[14,15]
		F2	CTCGAACTGCACCTCATGG	492–510	
		R1	CTTTACCAGCACGTGCTAGAAGG	896–874	
		R2	CAGAAGTTGTTATCGACATAGC	837–816	
		FS	ACCTCATGGTCATGTTATGG	502–521	
		RS	GACATAGCGAGTGTATGCC	823–805	
Yip-ORF1ab	China	F	ATGCATTTGCATCAGAGGCT	1866–1885	[17]
		R	TTGTTATAGCGGCCTTCTGT	1970–1951	
Pasteur-ORF1ab-1	France	F	ATGAGCTTAGTCCTGTTG	12,690–12,707	[15]
		P	AGATGTCTTGTGCTGCCGGTA	12,717–12,737	
		R	CTCCCTTTGTTGTGTTGT	12,797–12,780	
CN-CDC-ORF1ab	China	F	CCCTGTGGGTTTTACACTTAA	13,342–13,362	[13,15]
		P	CCGTCTGCGGTATGTGGAAAGGTTATGG	13,377–13,404	
		R	ACGATTGTGCATCAGCTGA	13,460–13,442	
Pasteur-ORF1ab-2	France	F	GGTAACTGGTATGATTTCG	14,080–14,098	[15]
		P	TCATACAAACCACGCCAGG	14,123–14,105	
		R	CTGGTCAAGGTTAATATAGG	14,186–14,167	
Young-ORF1ab	Singapore	F	TCATTGTTAATGCCTATATTAACC	14,155–14,178	[18]
		P	AACTGCAGAGTCACATGTTGACA	14,193–14,215	
		R	CACTTAATGTAAGGCTTTGTTAAG	14,243–14,220	
Corman-ORF1ab	Germany	F	GTGARATGGTCATGTGTGGCGG	15,431–15,452	[12]
		P1	CAGGTGGAACCTCATCAGGAGATGC	15,470–15,494	
		P2	CCAGGTGGWACRTCATCMGGTGATGC	15,469–15,494	
		R	CARATGTTAAASACACTATTAGCATA	15,530–15,505	
Won-ORF1ab	South Korea	F	CATGTGTGGCGGTTCACTAT	15,441–15,460	[16]
		R	TGCATTAACATTGGCCGTGA	15,558–15,539	
Chan-ORF1ab	China	F	CGCATACAGTCTTRCAGGCT	16,220–16,239	[10]
		P	TTAAGATGTGGTGCTTGCATACGTAGAC	16,272–16,303	
		R	GTGTGATGTTGAWATGACATGGTC	16,353–16,330	
HKU-ORF1ab	Hong Kong	F	TGGGGYTTTACRGGTAACCT	18,778–18,797	[11,15]
		P	TAGTTGTGATGCWATCATGACTAG	18,849–18,872	
		R	AACRCGCTTAACAAAGCACTC	18,909–18,889	
**S**					
Young-S	Singapore	F	TATACATGTCTCTGGGACCA	21,763–21,782	[18]
		P	CTAAGAGGTTTGATAACCCTGTCCTACC	21,789–21,816	
		R	ATCCAGCCTCTTATTATGTTAGAC	21,876–21,853	
Chan-S	China	F	CCTACTAAATTAAATGATCTCTGCTTTACT	22,712–22,741	[10]
		P	CGCTCCAGGGCAAACTGGAAAG	22,792–22,813	
		R	CAAGCTATAACGCAGCCTGTA	22,869–22,849	
Won-S	South Korea	F	CTACATGCACCAGCAACTGT	23,114–23,133	[16]
		R	CACCTGTGCCTGTTAAACCA	23,213–23,194	
NIID-JP-S	Japan	F1	TTGGCAAAATTCAAGACTCACTTT	24,354–24,377	[14,15]
		F2	TCAAGACTCACTTTCTTCCAC	24,364–24,384	
		R1	TGTGGTTCATAAAAATTCCTTTGTG	24,900–24,876	
		R2	ATTTGAAACAAAGACACCTTCAC	24,856–24,834	
		FS	AAGACTCACTTTCTTCCACAG	24,366–24,386	
		RS	CAAAGACACCTTCACGAGG	24,848–24,830	
**E**					
Won-E	South Korea	F	TTCGGAAGAGACAGGTACGTT	26,259–26,279	[16]
		R	CACACAATCGATGCGCAGTA	26,365–26,346	
Corman-E	Germany	F	ACAGGTACGTTAATAGTTAATAGCGT	26,269–26,294	[12]
		P	ACACTAGCCATCCTTACTGCGCTTCG	26,332–26,357	
		R	ATATTGCAGCAGTACGCACACA	26,381–26,360	
Huang-E	China	F	ACTTCTTTTTCTTGCTTTCGTGGT	26,295–26,318	[1]
		P	CTAGTTACACTAGCCATCCTTACTGC	26,326–26,351	
		R	GCAGCAGTACGCACACAATC	26,376–26,357	
**N**					
US-CDC-N-1	United States	F	GACCCCAAAATCAGCGAAAT	28,287–28,306	[9,15]
		P	ACCCCGCATTACGTTTGGTGGACC	28,309–28,332	
		R	TCTGGTTACTGCCAGTTGAATCTG	28,358–28,335	
NIH-TH-N	Thailand	F	CGTTTGGTGGACCCTCAGAT	28,320–28,339	[15]
		P	CAACTGGCAGTAACCA	28,341–28,356	
		R	CCCCACTGCGTTCTCCATT	28,376–28,358	
Young-N	Singapore	F	CTCAGTCCAAGATGGTATTTCT	28,583–28,604	[18]
		P	ACCTAGGAACTGGCCCAGAAGCT	28,608–28,630	
		R	AGCACCATAGGGAAGTCC	28,648–28,631	
US-CDC-N-3	United States	F	GGGAGCCTTGAATACACCAAAA	28,681–28,702	[9,15]
		P	AYCACATTGGCACCCGCAATCCTG	28,704–28,727	
		R	TGTAGCACGATTGCAGCATTG	28,752–28,732	
Corman-N	Germany	F	CACATTGGCACCCGCAATC	28,706–28,724	[12]
		P	ACTTCCTCAAGGAACAACATTGCCA	28,753–28,777	
		R	GAGGAACGAGAAGAGGCTTG	28,833–28,814	
Won-N	South Korea	F	CAATGCTGCAATCGTGCTAC	28,732–28,751	[16]
		R	GTTGCGACTACGTGATGAGG	28,849–28,830	
CN-CDC-N	China	F	GGGGAACTTCTCCTGCTAGAAT	28,881–28,902	[13,15]
		P	TTGCTGCTGCTTGACAGATT	28,934–28,953	
		R	CAGACATTTTGCTCTCAAGCTG	28,979–28,958	
NIID-JP-N	Japan	F	AAATTTTGGGGACCAGGAAC	29,125–29,144	[14,15]
		P	ATGTCGCGCATTGGCATGGA	29,222–29,241	
		R	TGGCAGCTGTGTAGGTCAAC	29,282–29,263	
		R-v3	TGGCACCTGTGTAGGTCAAC	29,282–29,263	
HKU-N	Hong Kong	F	TAATCAGACAAGGAACTGATTA	29,145–29,166	[11,15]
		P	GCAAATTGTGCAATTTGCGG	29,196–29,177	
		R	CGAAGGTGTGACTTCCATG	29,254–29,236	
US-CDC-N-2	United States	F	TTACAAACATTGGCCGCAAA	29,164–29,183	[9,15]
		P	ACAATTTGCCCCCAGCGCTTCAG	29,188–29,210	
		R	GCGCGACATTCCGAAGAA	29,230–29,213	
Chan-N	China	F	GCGTTCTTCGGAATGTCG	29,210–29,227	[10]
		P	AACGTGGTTGACCTACACAGST	29,257–29,278	
		R	TTGGATCTTTGTCATCCAATTTG	29,306–29,284	

Abbreviations: ORF1ab, open reading frame 1ab; S, spike; E, envelope; N, nucleocapsid; NIID-JP, National Institute of Infectious Diseases—Japan; CN-CDC, Chinese Center for Disease Control and Prevention; HKU, The University of Hong Kong; US-CDC, United States Centers for Disease Control and Prevention; NIH-TH, National Institute of Health of Thailand; F, forward; P, probe; R, reverse; FS, forward primer for sequencing; RS, reverse primer for sequencing.

**Table 3:** Summary of mismatches and suggested modifications to the oligos targeting animal SARS-CoV-2. Modifications to the oligo sequences (blue underlined) were performed only for mutations above the 0.5% threshold (present in four or more of the total genomes, red underlined). No modifications were suggested for mutations below the threshold (red). Deletions in the oligo targets are represented by underlined dashes, and each dash corresponds to a nucleotide that has been deleted.

Assay	Oligo	Sequence (5’-3’)	Mismatch Sequence(s) and Frequency	Mismatch Genomic Position	Suggested Modifications
NIID-JP-ORF1ab	FS	ACCTCATGGTCATGTTATGG	ACCTCATGGTCATG $\underline{---}$ TGG (409/787) ACCTCATGGTCA$\underline{C}$GTTATGG (40/787) ACCTCATGGTCACGTTATAG (1/787)	516–518 514 514, 520	Design new primers outside this region.
	RS	GACATAGCGAGTGTATGCC	GGCATATACTCGCTATGTC (4/791)	811	GACATAGCGAGT$\underline{R}$TATGCC
Yip-ORF1ab	R	TTGTTATAGCGGCCTTCTGT	ACRGAAGGCCGCTATAACAA (1/780) ACAGAAGGCCGCTGTAACAA (1/780) ACA$\underline{A}$AAGGCCGCTATAACAA (4/780)	1954 1964 1955	TTGTTATAGCGGCCTT$\underline{Y}$TGT
Pasteur-ORF1ab-1	R	CTCCCTTTGTTGTGTTGT	ACAACACAACAAAGG$\underline{A}$AG (149/792) ACAACACAACAAAG$\underline{A}$GAG (61/792)	12,795 12,794	CT$\underline{YY}$CTTTGTTGTGTTGT
CN-CDC-ORF1ab	F	CCCTGTGGGTTTTACACTTAA	CCCTGTGGGTTTTA$\underline{T}$ACTTAA (4/793)	13,356	CCCTGTGGGTTTTA$\underline{Y}$ACTTAA
Corman-ORF1ab	P2	CCAGGTGGWACRTCATCMGGTGATGC	CCAGGTGGAACCTCATCAGG$\underline{A}$GATGC (793/793)	15,480, 15,489	P2 was designed to detect SARS-CoV-2, SARS-CoV, and bat-SARS-related CoVs. For perfect match, use the other probe (probe1) of Corman-ORF1ab assay [12].
	R	CARATGTTAAASACACTATTAGCATA	TATGCTAATAGTGT$\underline{T}$TTTAACATTTG (793/793)	15,519	CARATGTTAAA$\underline{A}$ACACTATTAGCATA
Chan-ORF1ab	R	GTGTGATGTTGAWATGACATGGTC	GACCATGTCATATCAACATCACA$\underline{T}$ (792/792)	16,353	$\underline{A}$TGTGATGTTGAWATGACATGGTC
HKU-ORF1ab	P	TAGTTGTGATGCWATCATGACTAG	TAGTTGTGAT$\underline{T}$CAATCATGACTAG (12/793)	18,859	TAGTTGTGAT$\underline{K}$CWATCATGACTAG
Young-S	F	TATACATGTCTCTGGGACCA	TA $\underline{------}$ TCTCTGGGACCA (415/789)	21,765–21,770	Design new primers outside this region.
	R	ATCCAGCCTCTTATTATGTTAGAC	GTCTAA$\underline{T}$ATAATAAGAGGCTGGAT (10/790)	21,859	GTCTAA$\underline{Y}$ATAATAAGAGGCTGGAT
NIID-JP-S	FS	AAGACTCACTTTCTTCCACAG	AAGACTCACTTTTTTCCACAG (3/790) AAGACTCACTTTCTTCCACAT (1/790)	24,378 24,386	Individual mutations are below the threshold. No modifications are currently required.
Won-E	R	CACACAATCGATGCGCAGTA	TACTGCGC$\underline{T}$TCGATTGTGTG (775/775)	26,354	CACACAATCGA$\underline{A}$GCGCAGTA
US-CDC-N-1	P	ACCCCGCATTACGTTTGGTGGACC	ACCTCGCATTACGTTTGGTGGACC (1/792) ACCCCGCATTACGTTTGTTGGACC (1/792) AC$\underline{T}$CCGCATTACGTTTGGTGGACC (5/792)	28,312 28,326 28,311	AC$\underline{Y}$CCGCATTACGTTTGGTGGACC
	R	TCTGGTTACTGCCAGTTGAATCTG	CAGATTCAACTGGCAGTAACCAG$\underline{C}$ (95/792) CAGATTCAACTGGCAGTAAACAGA (1/792)	28,358 28,354	$\underline{K}$CTGGTTACTGCCAGTTGAATCTG
NIH-TH-N	R	CCCCACTGCGTTCTCCATT	$\underline{C}$ATGGAGAACGCAGTGGGG (95/792)	28,358	CCCCACTGCGTTCTCCAT$\underline{K}$
Young-N	P	ACCTAGGAACTGGCCCAGAAGCT	ACCTAGGAACTGG$\underline{G}$CCAGAAGCT (793/793)	28,621	ACCTAGGAACTGG$\underline{G}$CCAGAAGCT
CN-CDC-N	F	GGGGAACTTCTCCTGCTAGAAT	$\underline{AAC}$GAACTTCTCCTGCTAGAAT (446/785) $\underline{AACT}$AACTTCTCCTGCTAGAAT (13/785)	28,881–28,884	$\underline{RRSK}$AACTTCTCCTGCTAGAATor design new primer
NIID-JP-N	R	TGGCAGCTGTGTAGGTCAAC	GTTGACCTACACAG$\underline{G}$TGCCA (789/789)	29,277	This mismatch is already corrected in R-v3 primer of NIID-JP-N assay [14].
Chan-N	R	TTGGATCTTTGTCATCCAATTTG	CAAATTGGATGACAAA$\underline{T}$ATCCAA (12/789) CAAATTGGATTACAAAGATCCAA (1/789)	29,300 29,294	TTGGAT$\underline{M}$TTTGTCATCCAATTTG

Abbreviations: ORF1ab, open reading frame 1ab; S, spike; E, envelope; N, nucleocapsid; NIID-JP, National Institute of Infectious Diseases—Japan; CN-CDC, Chinese Center for Disease Control and Prevention; HKU, The University of Hong Kong; US-CDC, United States Centers for Disease Control and Prevention; NIH-TH, National Institute of Health of Thailand; F, forward; P, probe; R, reverse; FS, forward primer for sequencing; RS, reverse primer for sequencing.

## Data Availability

All data generated and/or analyzed in this study are present in the article and supplementary files.

## Supplementary Materials

The following are available online at https://www.mdpi.com/2076-0817/10/3/371/s1, Table S1: Information on SARS-CoV-2 genomes used in the current study including the virus isolate, accession number, host, geographic region or country, genome length, collection date, database from which they were downloaded, and the percentage of ambiguous bases (%N), Table S2: Results of the bioinformatic analysis of 28 diagnostic PCR targets using 793 animal SARS-CoV-2 genomes.
